# Supplementary material for: A Prospective, Multicenter, Randomized, Noninferiority Trial of Stopad® Versus Tachosil® for Hemostasis After Liver Resection
Source: Cancers (Basel). 2025 Feb 23;17(5):757. doi: 10.3390/cancers17050757 (PMC11898775; doi:10.3390/cancers17050757)
Supplement: Supplementary file 1 [file cancers-17-00757-s001.zip › cancers-3433407-supplementary.pdf]

**Supplementary Table S1. List of subjects who experienced serious adverse event (SAE)**

| Subject ID | Institution | Treatment Group | Sex | Age | Type of SAE | Duration of SAE (d) | Severity |
|---|---|---|---|---|---|---|---|
| 01S-10 | SH | Tachosil | M | 67 | Hematoma | 6 | Moderate |
| 01S-17 | SH | Stopad | M | 68 | Wound evisceration | 1 | Moderate |
| 01S-24 | SH | Stopad | M | 62 | Vomiting | 9 | Mild |
| 03S-03 | SNUBH | Tachosil | M | 66 | Portal vein thrombosis | 3 | Mild |
| 03S-06 | SNUBH | Stopad | M | 48 | Perihepatic fluid collection | 5 | Moderate |
| 03S-08 | SNUBH | Stopad | M | 54 | Portal vein thrombosis | 8 | Moderate |
| 03S-12 | SNUBH | Tachosil | M | 64 | Biliary fistula | 14 | Moderate |
| 03S-27 | SNUBH | Stopad | F | 64 | Respiratory failure | 5 | Moderate |
| 03S-29 | SNUBH | Stopad | M | 68 | Ileus | 15 | Mild |
| 03S-35 | SNUBH | Stopad | M | 65 | Ileus | 8 | Mild |
| 04S-03 | GSH | Tachosil | M | 58 | Upper gastrointestinal bleeding | 7 | Moderate |
| 04S-16 | GSH | Stopad | F | 83 | Nausea | 4 | Mild |
| 04S-23 | GSH | Stopad | F | 47 | Adrenal hemorrhage | 7 | Moderate |
| 04S-34 | GSH | Stopad | M | 75 | Ileostomy site bleeding | 8 | Mild |

Abbreviations: SAE, serious adverse event; SH, Severance Hospital; SNUBH: Seoul National University Bundang Hospital; GSH: Gangnam Severance Hospital

Note: All SAEs were classified as definitely not related to the experimental or control products, and all patients with SAE recovered without sequelae.
